# Heterogeneous rates of genome rearrangement contributed to the disparity of species richness in Ascomycota

**DOI:** 10.1186/s12864-018-4683-0

**Published:** 2018-04-24

**Authors:** Ahmad Rajeh, Jie Lv, Zhenguo Lin

**Affiliations:** 1 Department of Biology, Saint Louis University, St. Louis, MO 63103 USA; 2 Department of Computer Science, Saint Louis University, St. Louis, MO 63103 USA; 3 Department of BioSciences, Rice University, Houston, TX 77005 USA

**Keywords:** Chromosomal rearrangements, Species richness, Ascomycota, Taphrinomycotina, Pezizomycotina, Saccharomycotina

## Abstract

**Background:**

Chromosomal rearrangements have been shown to facilitate speciation through creating a barrier of gene flow. However, it is not known whether heterogeneous rates of chromosomal rearrangement at the genome scale contributed to the huge disparity of species richness among different groups of organisms, which is one of the most remarkable and pervasive patterns on Earth. The largest fungal phylum Ascomycota is an ideal study system to address this question because it comprises three subphyla (Saccharomycotina, Taphrinomycotina, and Pezizomycotina) whose species numbers differ by two orders of magnitude (59,000, 1000, and 150 respectively).

**Results:**

We quantified rates of genome rearrangement for 71 Ascomycota species that have well-assembled genomes. The rates of inter-species genome rearrangement, which were inferred based on the divergence rates of gene order, are positively correlated with species richness at both ranks of subphylum and class in Ascomycota. This finding is further supported by our quantification of intra-species rearrangement rates based on paired-end genome sequencing data of 216 strains from three representative species, suggesting a difference of intrinsic genome instability among Ascomycota lineages. Our data also show that different rates of imbalanced rearrangements, such as deletions, are a major contributor to the heterogenous rearrangement rates.

**Conclusions:**

Various lines of evidence in this study support that a higher rate of rearrangement at the genome scale might have accelerated the speciation process and increased species richness during the evolution of Ascomycota species. Our findings provide a plausible explanation for the species disparity among Ascomycota lineages, which will be valuable to unravel the underlying causes for the huge disparity of species richness in various taxonomic groups.

**Electronic supplementary material:**

The online version of this article (10.1186/s12864-018-4683-0) contains supplementary material, which is available to authorized users.

## Background

Chromosomal rearrangements, such as translocation, inversion, duplication or deletion events, have profound effects on organismal phenotype through impacting gene expression and disrupting the function of genes [1]. It is a long-held view that chromosomal rearrangements are generally deleterious [2]. Many studies found that chromosomal rearrangements reduced gene flow between populations in a wide range of taxonomic groups, such as sunflowers [3, 4], oilseed rape (*Brassica napus*) [5], fruit flies [6], shrews [7], mosquitoes [8], house mouse [9] and yeasts [10–13]. For example, crosses between different natural isolates of fission yeast *Schizosaccharomyces pombe* with different karyotypes displayed significantly lower hybrid viability than those with similar karyotypes [12]. Other studies also supported that chromosomal translocation is an important contributor to the yeast speciation process [11, 14, 15]. Therefore, the chromosomal speciation theory proposed that chromosomal rearrangements contribute to the speciation process through restricting gene flow between populations [16–20]. Two main models (hybrid-sterility models and suppressed recombination models) have been proposed to explain the mechanisms of chromosomal rearrangements in the process of speciation [21]. A natural question following the chromosomal speciation theory is whether the rates of chromosomal rearrangement at a genome-scale correlate with the rates of speciation, or species richness, among different groups of organisms. The huge disparity in species richness across the tree of life is one of the most remarkable and pervasive patterns on Earth [22]. Some groups, like beetles and flowering plants, are well-known for their enormous species diversity, while most other groups contain far fewer species [23]. It has been proposed that the species richness of a lineage depends on the interplay between evolutionary and ecological processes [24], such as ages of clades [25], net diversification rates (speciation minus extinction) [26], or ecological limits [27]. However, the impact of different rates of genome rearrangement in the formation of species richness disparity has not been systematically investigated.

Compared to the animals and plants, the fungal phylum Ascomycota can serve as an ideal system to study the connection between the rates of genome rearrangement and disparity of species richness. Ascomycota is one of the most diverse and ubiquitous phyla of eukaryotes with ~ 64,000 known species that accounts for approximately 75% of all described fungi [28]. Ascomycota comprises three subphyla (or subdivisions): Saccharomycotina (e.g., *Saccharomyces*, *Pichia*, *Candida*), Taphrinomycotina (e.g., *Schizosaccharomyces, Pneumocystis*), and Pezizomycotina (e.g., *Aspergillus*, *Neurospora*, *Peziza*) [29]. The species numbers of the three Ascomycota subphyla differ by at least two orders of magnitude. Pezizomycotina is the most species-rich subphylum, comprising nearly 59,000 known species [28]. Saccharomycotina contains ~ 1000 known species that are distributed in 12 families [30]. In contrast, Taphrinomycotina includes only six genera and 150 species [31]. Because the three subphyla have similar ages, which is ~ 500 million years [32], the huge disparity of species richness among them appears to be due to non-age factors, which remains to be elucidated.

The genomes of many Ascomycota species have been sequenced and well assembled, which make it possible to investigate the rates of genome rearrangement in each subphylum and to determine whether they are associated with the disparity in species richness. In addition, at least one well-studied model organism can be found in each Ascomycota subphylum, such as the budding yeast *Saccharomyces cerevisiae* of Saccharomycotina, *Sch. pombe* of Taphrinomycotina and *Neurospora crassa* of Pezizomycotina. The genomes of many populations or strains of the three species have been sequenced by Illumina paired-end sequencing, which can be used to quantify the rates of genome rearrangement under much smaller evolutionary timescales [33–36]. The rates of genome rearrangement inferred between different species and within a species can provide reliable measurements of genome instability and, together, provide the opportunity to test the correlation between genome instability and species richness. In this study, we used genomes of 71 Ascomycota species to estimate the rates of genome rearrangement between different species in each subphylum and used paired-end sequencing data from 216 strains to calculate rates of genome rearrangement within a species for the three model organisms. We found that the rates of genome rearrangement are positively correlated with species richness at both ranks of subphylum and class. Therefore, our study provides the first genome-scale evidence to support an important role of genome rearrangement in promoting species richness, and suggests that different rates of genome rearrangement at least partly explain the species richness disparity among different Ascomycota lineages. Our findings also provide a new direction in investigating the underlying causes for the disparity of species richness in many other lineages of organisms, such as insects, fishes, and flowering plants.

## Results

### Inference of orthologous groups and evolutionary history of Ascomycota species examined

Chromosomal rearrangement events inevitably change the order of genes on a chromosome. Therefore, the degree of gene order divergence (GOD) reflects the rate of chromosomal rearrangement [37]. Using GOD also allows us to measure the degree of genome rearrangement between evolutionarily distantly-related species [38]. Considering that the divergence times between many species examined in this study may exceed 300 million years [32], using GOD to estimate the degree of genome rearrangement between species is a reasonable and feasible approach. Inference of GOD between two species requires accurate annotation of gene location in the genome and identification of orthologous genes. To provide an accurate estimation of rates of genome rearrangement, we only used genomes that are well-assembled (supercontigs < 50) and annotated (with complete coordination annotation of protein-coding sequences). A total number of 71 genomes that include 39 Pezizomycotina species, 27 Saccharomycotina species, and 5 Taphrinomycotina meet the above criteria and were retrieved from NCBI RefSeq database for our subsequent analyses (Additional file 1: Table S1). Orthologous groups between every pair of species were identified using InParanoid [39].

**Fig. 1:**
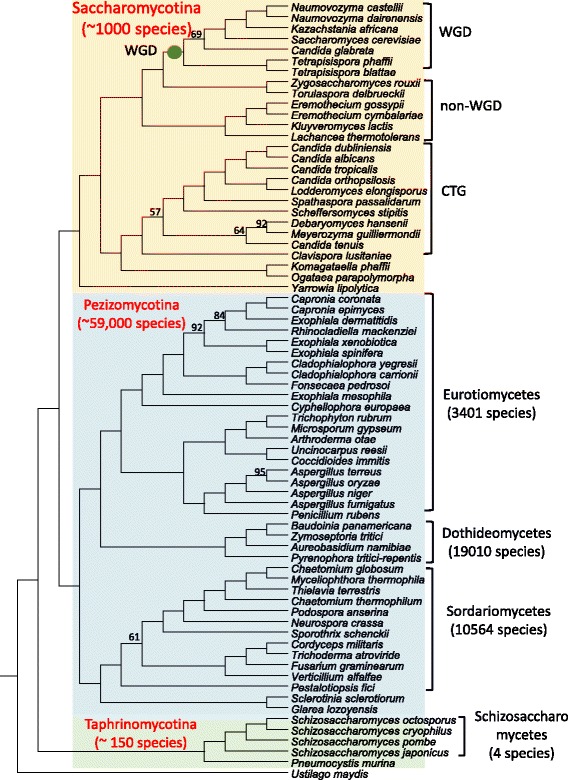
Phylogenetic relationships among 71 Ascomycota species examined. The phylogenetic relationships were inferred from coalescence-based analysis of 160 orthologous gene sets. A Basidiomycota species *Ustilago maydis* was used as an outgroup. Only bootstrap support values < 100 are shown. The branch is not drawn to scale. The species numbers of major clades were obtained from [28]. The green dot indicates the occurrence of whole genome duplication (WGD)

To infer the evolutionary relationships for the 71 Ascomycota species examined, we reconstructed a species phylogenetic tree through coalescent-based phylogenetic analyses using one-to-one orthologous groups (see Methods). A Basidiomycota species *Ustilago maydis* was included as an outgroup for species phylogeny inference. A total number of 160 one-to-one orthologous groups (Additional file 2: Table S2) were identified using InParanoid [39]. Three major monophyletic groups which are corresponding to the three subphyla can be identified from the coalescent-based species tree (Fig. 1). The subphylum Taphrinomycotina appears to be the first lineage that had diverged from the other two subphyla, which is consistent with previous work [40].

A prerequisite to calculating the rates of genome rearrangement between two species is their divergence times. Due to lack of fossil records, the dating of divergence times between fungal species is difficult, and it is inconsistent among studies [41]. The divergence of protein sequences has been commonly used to represent the evolutionary divergence time between two species based on the assumption that the difference of amino acid sequences increases approximately linearly with time [42]. In addition, it is more accurate to estimate the divergence time between two species using sequence divergence level based on concatenation of many protein sequences than using a single sequence or the average distance for all proteins [43]. Therefore, to infer the evolutionary times of all species examined, we calculated the sequence distances using concatenated protein sequences of the 160 orthologous groups (see Methods, Additional file 3: Table S3).

### The relationships between gene order divergence and sequence distance in Ascomycota

We first estimated the degree of GOD between two species by calculating the proportion of gene orders or gene neighborhoods that are not conserved (*pGOD*), which was calculated by dividing the number of lost gene neighborhoods by all gene neighborhoods in the two species (see Methods). Within each subphylum, the values of *pGOD* vary greatly between different species pairs (Additional file 3: Table S3). Specifically, the *pGOD* values range from 0.03 to 0.796 between the 39 Pezizomycotina species, from 0.012 to 0.966 between the 27 Saccharomycotina species and 0.193 to 0.857 between the 5 Taphrinomycotina species. As the divergence times range from several to hundreds of million years between these species, it is expected to observe a wide range of variations in *pGOD* values. Considering that the conservation of gene order between the most distantly-related species within a subphylum is already close to nonexistent, we did not calculate the cross-subphyla gene order divergence.

**Fig. 2:**
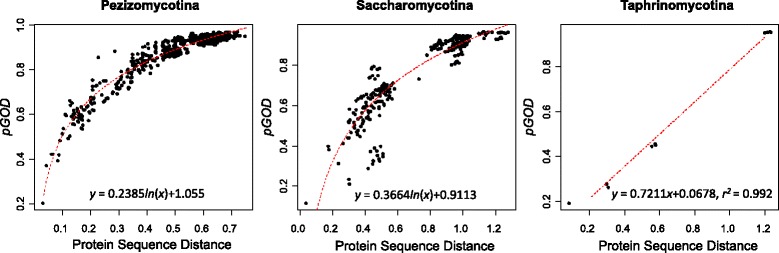
The correlation between gene order divergence (*pGOD*) and sequence distance in the three Ascomycota subphyla. Each dot represents a pair of species compared. Protein sequence distance was measured based on concatenating protein sequence alignments of 160 orthologous groups

To infer the relationships between *pGOD* values and divergence times, we plotted *pGOD* values against their corresponding sequence distances which were calculated based on the 160 concatenated protein sequences. It is a general pattern that *pGOD* values increase with the increase of sequence distance (Fig. 2). However, the trend of increase is different among the three subphyla. In Pezizomycotina and Saccharomycotina, we observed a non-linear correlation between *pGOD* and sequence distance. The increase of *pGOD* plateaus when sequence distance is large, which is an indication of saturation of *pGOD*. Such patterns can be fitted by a logarithmic regression model: *y* = 0.236ln(*x*) + 1.055 in Pezizomycotina, and *y* = 0.366ln(*x*) + 0.911 in Saccharomycotina. In contrast, *pGOD* values in Taphrinomycotina form a linear correlation with sequence distance (*y* = 0.7211*×* + 0.0678, *r*^*2*^ = 0.992). Based on the three regression models, the sequence distance to lose 50% of gene order, or gene order half-life, is 0.095 in Pezizomycotina, 0.325 in Saccharomycotina and 0.599 in Taphrinomycotina. If we use sequence distance as a proxy for divergence time, the gene order half-life of Pezizomycotina species is ~ 3.4× shorter than Saccharomycotina species, and is ~ 6.3× shorter than Taphrinomycotina species. Therefore, the large differences of gene order half-life indicate the divergence rates of gene order are heterogeneous rates among the three Ascomycota subphyla, and species-rich lineage has a much short gene order half-life than species-poor lineage.

### Rates of genome rearrangement correlate with species richness among Ascomycota subphyla

The saturation of accumulation of gene order divergence in Pezizomycotina and Saccharomycotina suggests that multiple breakages of a gene neighborhood may have occurred between distantly related species. Therefore, the degree of GOD could be underestimated, particularly for distantly related species, if multiple breakages of a gene neighborhood are not considered. If we assume for simplicity that the rates of gene order loss are the same for all neighborhoods, the probability of the number of loss events at a given gene neighborhood follows the Poisson distribution [44]. However, this assumption does not hold because significant variations of *pGOD* among different chromosomal regions were observed in all subphyla based on our sliding-window analysis of gene order divergence (Additional file 4: Figure S1). Therefore, a correction model also needs to take into consideration the variation of *pGOD* across different chromosomal regions, similar to the variation of amino acid substitutions. It has been recognized that the gamma distribution can effectively model the realistic variation in mutation rates of molecular sequences [45]. Therefore, we can apply the gamma distribution to estimate the degree of GOD, called here gamma distance of GOD (*dGOD*). The shape or gamma parameter *α*, was estimated based on the distributions of *pGOD* values across different chromosomal regions. Three model organisms (*S. cerevisiae*, *N. crassa,* and *Sch. pombe*) were used as representative species to estimate the *α* parameter for each subphylum (see Methods). The values of the *α* parameter values were relatively consistent among different comparisons and subphyla, ranging from 2.29 to 3.86 (Additional file 6: Table S4). The median *α* parameter values of each species (*N. crassa*: 2.83*, S. cerevisiae*: 2.69*, Sch. pombe*: 3.10) were used to calculate *dGOD* values for each subphylum.

**Fig. 3:**
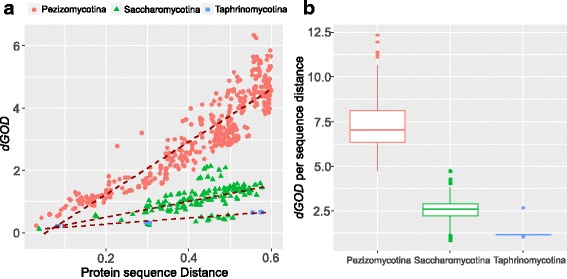
Heterogenous rates of gene order divergence among Ascomycota subphyla. **a** A lineage correlation between gamma distance of gene order divergence (*dGOD*) and sequence distance in all three subphyla. **b** Boxplot showing the different rates of *dGOD* among the three Ascomycota subphyla. The rate of *dGOD* was calculated as *dGOD* per unit of protein sequence distance

In addition, because the variance of *dGOD* increases with the increase of gene order divergence, the *dGOD* for distantly related species may be inaccurate. Therefore, we only included species pairs with sequence distance < 0.6, which comprises most species examined within each class of Ascomycota. By plotting the *dGOD* values against their sequence distance, we found that the *dGOD* values correlate linearly with sequence distance in all three subphyla (Fig. 3a). Based on the linear regression model, the rate of genome rearrangement in Pezizomycotina (*y* = 8.40*×* - 0.44, *r*^*2*^ = 0.84) is 3.31× higher than Saccharomycotina species (*y* = 2.54× - 0.001, *r*^*2*^ = 0.30), and is 8.48× higher than Taphrinomycotina (*y* = 0.99*×* + 0.086, *r*^*2*^ = 0.96), which is similar to the results based on gene order half-life.

To quantify the degree of GOD per unit of divergence time for each subphylum, we normalized the *dGOD* by sequence distance for each pair of species compared. Highly heterogeneous rates of *dGOD* were detected among the three groups (ANOVA one-way test, *p* < 0.001, Fig. 3b). The average *dGOD* per genetic distance in Pezizomycotina is 7.26 ± 1.32, which is significantly higher than that of Saccharomycotina (2.54 ± 0.79, *p* < 0.001, Tukey post hoc test). The average *dGOD* per genetic distance in Saccharomycotina is also significantly higher than that of Taphrinomycotina (1.40 ± 0.57, *p* < 0.001), supporting a positive correlation between rates of genome rearrangement and species richness among the three subphyla of Ascomycota.

### Rates of genome rearrangement positively correlated with species richness at the rank of class

**Fig. 4:**
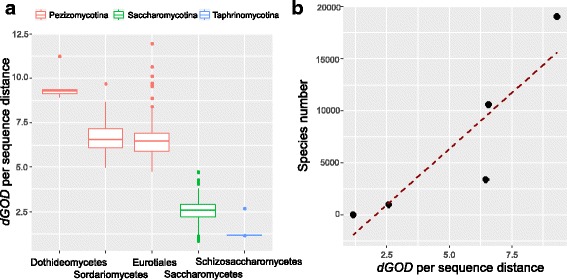
Heterogeneous rates of gene order divergence within subphylum. **a**. Rates of genome rearrangement positively correlate with species richness at the level of class in Ascomycota. The rates of genome rearrangement were calculated as *dGOD* per unit of protein sequence distance. **b**. A scatter plot of the species number and median value of *dGOD* per unit of protein sequence distance in the five Ascomycota classes. A positive correlation can be observed between the two variables (Pearson correlation coefficient *r* = 0.89)

Our data support a strong correlation between rearrangement and species richness at the rank of subphylum level in Ascomycota. To determine if the same pattern also presents at lower taxonomic ranks, we compared the rearrangement rates between different classes of Ascomycota species. To reduce the potential impact of small sample size, we only compared classes with at least four species examined in this study. In Pezizomycotina, three classes meet the threshold, which are Eurotiomycetes, Sordariomycetes and Dothideomycetes (Fig. 1, and Additional file 1: Table S1). The numbers of documented species in the three Pezizomycotina classes are 3400, 10,564, and 19,010 respectively [28]. All the Saccharomycotina species examined belong to the only class of this subphylum Saccharomycetes, which comprises ~ 1000 known species [30]. In Taphrinomycotina, only the class of Schizosaccharomycetes meet the criteria. Only four species (*Schizosaccharomyces pombe*, *Sch. japonicus*, *Sch. octosporus* and *Sch. cryophilus*) have been described in Schizosaccharomycetes [46]. It was suggested the Schizosaccharomycetes diverged from other Taphrinomycotina lineages nearly 500 MYA [46], indicating extremely limited species diversity. As shown in Fig. 4a, the most species-rich class, Dothideomycetes has the highest rearrangement rate among all classes examined, while the most species-poor class, Schizosaccharomycetes has the lowest rearrangement rate. By plotting the number of species against median rates of rearrangement of all classes (Fig. 4b), a significant positive correlation can be observed between the two variables (Pearson correlation coefficient *r* = 0.89), supporting that the rearrangement rates are also strongly correlated with species richness at the class level in Ascomycota.

### The impacts of whole genome duplication and lifestyle on rates of genome rearrangement

**Fig. 5:**
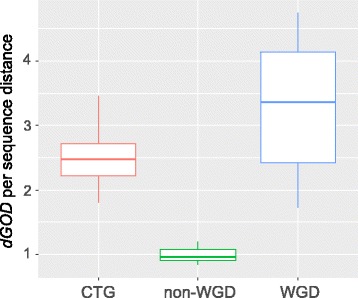
Heterogeneous rates of gene order divergence in the class of Saccharomycetes. The rates of genome rearrangement were calculated as *dGOD* per unit of protein sequence distance. The Saccharomycetes species that have experienced an ancient whole genome duplication have higher rates of genome rearrangement than the CTG group and non-WGD group

The scatter plot of *dGOD* against sequence distance shows that the rates of gene order divergence have noticeable variations among Saccharomycetes species (Fig. 3a), which is consistent with a previous study [37]. To infer other factors that might influence the rearrangement rates in Saccharomycetes, we further divided the Saccharomycetes species examined into different groups based on their evolutionary relationships. Two monophyletic clades with more than four species can be identified from the species tree in Fig. 1. One of them includes many pathogenic yeast *Candida* species and as well as non-pathogenic yeast *Debaryomyces hansenii*, which is the co-called CTG group because of the reassignment of the CUG codon [47]. The second monophyletic clade, which includes the model organisms *S. cerevisiae*, belongs to the *Saccharomyces* complex [48]. The *Saccharomyces* complex has experienced a whole genome duplication (WGD) about 100 MYA [49, 50]. Previous studies have shown that extensive genome rearrangement events have shaped the yeasts’ genomes since WGD [51, 52]. Therefore, we divided the *Saccharomyces* complex into two groups: WGD and non-WGD, to better understand the impact of WGD on genome stability. In terms of divergence rate of gene order (Fig. 5), the WGD group is significantly higher than the other two groups (*p* < 0.001), while the CTG group has a much higher rate of *dGOD* than the non-WGD group. Therefore, our results support that whole genome duplication, as well as pathogenic lifestyle, may have elevated the rates of rearrangement, which is consistent with previous studies in *Candida albicans* [37] and pathogenic bacteria [53].

### Imbalanced rearrangement as an important contributor to the heterogeneous rates of genome rearrangement

**Fig. 6:**
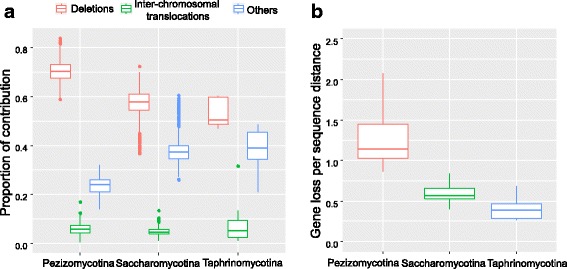
Gene loss as a major contributor to the heterogeneous rates of genome rearrangement among Ascomycota subphyla. **a** Boxplot showing the proportion of three types of rearrangements that contribute to gene order divergence in each subphylum. **b** Ascomycota sequences have the highest rates of gene loss per sequence distance among the three subphyla, while Taphrinomycotina species have the lowest rate. The outliers are not drawn in B for better readability

The gene order can be changed by both types of genome rearrangement, balanced and imbalanced. Unlike balanced rearrangements (e.g., inversions and reciprocal translocations), the imbalanced rearrangements (deletions and duplications) also change the gene dosage or gene content due to gain or loss of gene copies. To better understand the underlying causes for the heterogeneous rates of arrangements, we estimated the relative contribution of different types of genome rearrangement in each subphylum. If loss of gene order between two species is due to the absence of one or two orthologous genes in the other species, we considered it as deletion or imbalanced rearrangement. If the orthologous genes of two neighboring genes are located on different chromosomes in the other species, we considered it as inter-chromosomal translocation. If the orthologous genes of two neighboring genes are located on the same chromosome but are not neighboring genes in the other species, it is likely due to other balanced rearrangements, such as inversion or intra-chromosomal transaction, which is defined as “Others” type. We quantified the contributions of the three types of rearrangements for all pairwise genome comparisons in each subphylum (Fig. 6a and Additional file 3: Table S3). In most cases, deletions account for over 50% of gene order divergence, suggesting that imbalanced rearrangements play a major role in genome instability. Furthermore, deletions have the more contributions for gene order divergence in Pezizomycotina, with an average of 70.5 ± 4.4%, more than 56.5 ± 6.67% in Saccharomycotina and 53.2 ± 5.85% in Taphrinomycotina. To infer if the increased contribution of deletion is due to a high rate of gene loss, we calculated the rate of gene loss per unit of sequence distance for each pairwise comparison. In Pezizomycotina, the average rate of gene loss is 1.37 ± 0.63 per unit of sequence distance, which is much higher than Saccharomycotina (0.61 ± 0.15) and Taphrinomycotina (0.39 ± 0.15) (Fig. 6b). Lineage-specific gene losses have been shown to have the largest effect in terms of lowering the meiotic fertility of hybrids between *Saccharomyces* sensu stricto species and other yeasts that have inherited the same genome duplication [54]. Therefore, the elevated rate of deletions or imbalanced rearrangements in Pezizomycotina species is an important factor for their higher rates of genome rearrangement.

### Pezizomycotina has the highest rearrangement rates within a species

The heterogeneous rates of genome rearrangement between different Ascomycota subphyla could be due to their different intrinsic genome instability, as well as the constraint of different environmental niches and lifestyle. As the divergence times of different populations within a species are much shorter than that between different species, the impacts of environmental constraint on the rates of genome rearrangement among populations are significantly reduced. Therefore, the rates of genome rearrangement between closely related strains or populations can be used to measure the intrinsic genome instability of a species. The genome rearrangement events between closely related organisms can be identified using paired-end mapping (PEM) based on high-quality paired-end sequencing data [33–36]. Because paired-end sequencing data of many strains are available in the three well-studied representative organisms: *S. cerevisiae* in Hemiascomycota, *Sch. pombe* in Taphrinomycotina and *N. crassa* in Pezizomycotina, they were used to obtain a reliable measurement of intrinsic genome instability for the three Ascomycota subphyla.

**Fig. 7:**
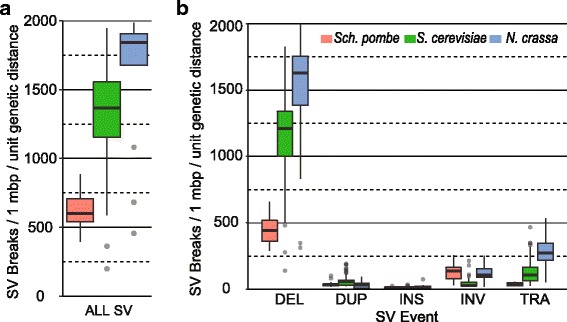
Different intra-species rates of genome rearrangement between three representative species. The structural variants (SVs) of each strain were identified based on Illumina paired-end sequencing reads and validated by local assembly. **a** Normalized density of SVs support the highest rates of intra-species rates of rearrangement in *N. crassa*. **b** Deletion is the most abundant SV in all three species. DEL: deletion; DUP: tandem duplication; INS: insertion; INV: inversion, TRA: translocation

We identified structural variants (SVs) based on Illumina paired-end reads by combining split-read, read-depth, and local-assembly evidence (see Methods). We identified 15,251 SVs from 29 *N. crassa* strains (525.90 SVs/strain), 13,647 SVs from 155 *S. cerevisiae* strains (88.05 SVs/strain) and 1218 SVs from 32 *Sch. pombe* strains (38.06 SVs/strain) (Additional file 7: Table S5 and Additional file 8: Table S6). Considering that the genome sizes of the three species are different (40 Mb in *N. crassa* and ~ 12 Mb in *S. cerevisiae* and *Sch. pombe*) (Additional file 7: Table S6), and the divergence times between strains could also be different, the rates of genome rearrangement between two strains need to normalize the numbers of SVs by its genome size and divergence time. As the divergence times between most strains are not available, we used their genetic distance as a proxy. The genetic distance was calculated as the frequency of single nucleotide polymorphisms (SNPs) based on their sequencing reads (see Methods). For each strain, we calculated the number of SV breakpoints per 1 million base pairs (Mbp) per unit genetic distance to infer its rate of intra-species genome rearrangement. Highly heterogeneous rates of intra-species genome rearrangement are observed among the three species (Fig. 6a). Specifically, *N. crassa* has a significantly faster intra-species genome rearrangement than *S. cerevisiae* (*p* < 0.001, Student’s T-test), and *S. cerevisiae* has a significantly faster genome rearrangement than *Sch. pombe* (*p* < 0.001). In addition, similar to the results of inter-species rearrangement, deletions account for the most of SVs between different strains in each species (Fig. 7b). Therefore, the patterns of intra-species genome rearrangement in the three subphyla is consistent with the inter-species gene order divergence, suggesting that the heterogeneous rates of genome rearrangement among the three Ascomycota subphyla are likely due to the difference of intrinsic genome instability.

### Transposable elements contributed differently to genome rearrangement between species

Transposable elements (TEs) have been shown to play a crucial role in genome shaping via recombination and expansion events, leading to chromosomal rearrangements and new gene neighborhoods [55–57]. In many pathogenic fungi, invasion and expansion of transposable elements have facilitated chromosomal rearrangements and gene duplications [57–59]. Recombination between transposable elements is a source of chromosomal rearrangements in the budding yeast *S. cerevisiae* [60]. Moreover, large genomic changes caused by transposons have been shown to contribute to rapid adaptation to changing environments [56]. Therefore, we investigated the contributions of TEs in the genomes of 216 strains examined. Most TEs found in fungal genomes belong to the Long Terminal Repeats (LTR) retrotransposons [61, 62]. Unlike animal and plant genomes, most fungal species have low TE contents. One hundred ninety complete LTR retrotransposons or LTR fragments were identified in *N. crassa*, which only account for 1.7% of its genome [62]. About 3% of the budding yeast *S. cerevisiae* genomes are transposable elements. In the fission yeast *Sch. pombe*, transposable elements only accounts for 1.18% of its genome. Massive loss of transposable elements was observed in three fission yeast genomes after their split from *Sch. japonicas* [46].

In *S. cerevisiae*, 8331 of 13,647 (61.1%) SVs were found within 100 bp of LTR retrotransposons or LTR fragments (Additional file 8: Table S6). Among them, 5585 SVs in *S. cerevisiae* are located within 100 bp of the 50 complete LTR retrotransposons, accounting for 40.9% of all SVs identified in the 155 *S. cerevisiae* strains. The substantial portion of SVs associated with LTRs in *S. cerevisiae* is consistent with a previous study based on a survey of spontaneous mutations [63]. In *Sch. pombe,* only 24.6% (300) SVs were found within 100 bp of LTRs. This number is further reduced to 1.47% (225 SVs) in *N. crassa*, suggesting TEs have contributed quite differently to the genome rearrangement among the three species. Therefore, TEs might play an important role in generating genome instability in *S. cerevisiae*, but its role is limited in the other fungal species, particularly in *N. crassa*. Furthermore, because the numbers of TEs are highly dynamic between different fungal species within a subphylum [62], it suggests that the number of TEs is probably not a leading factor for the heterogeneous rates of genome rearrangement among the three Ascomycota subphyla.

## Discussion

In this study, we found that the rates of genome rearrangements are highly heterogeneous among different lineages of fungal species and there is a positive correlation between the rates of genome rearrangement and species richness. These results offer a plausible explanation for the huge disparity of species richness among the three Ascomycota subphyla and between different classes. Therefore, our study extends the chromosomal theory of speciation to the genome-scale. Specifically, the level of chromosome rearrangement at the genome scale could impact species richness, providing a clue to study the underlying genetic basis of species richness variation among taxonomic groups. The species richness disparity is a pervasive phenomenon that is observed in many different lineages [23]. The underlying causes for the disparity of species richness in other lineages of organisms, such as insects, fishes, and flowering plants, remains to be elucidated. Here, we provided solid lines of evidence to support an important role of rates of genome rearrangement in promoting species richness. With rapid accumulations of genome sequencing data, it will soon become possible to determine the extent to which the heterogeneity of the rates of genome rearrangements contributed to the species richness disparity in those animal and plant lineages.

On the other hand, our study also raises some questions for future research. The first question is what major factors have resulted in the highly heterogeneous rates of chromosomal rearrangements among the three Ascomycota lineages? We showed here that the occurrence of whole genome duplication and pathogenic lifestyle might have elevated the gene order divergence and rates of genome rearrangements (Fig. 5). Nearly 90% of duplicate genes generated by WGD has lost after the occurrence of WGD [49, 50], which inevitably led to breakage of a large number of gene neighborhoods and increased the divergence of gene order. The pathogenic lifestyle of some species, such as *C. albicans* may have accumulated more rearrangements because of selective sweeps due to adaptation to narrow ecological niches, or less efficient selection due to smaller population size [37]. The rate of gene order divergence for the group of non-WGD and non-pathogenic budding yeast, such as *Kluyveromyces lactis* and *Zygosaccharomyces rouxii*, are not quite different from that of fission yeasts, supporting an influential impact of WGD and pathogenic lifestyle on the genome stability. Recombination between non-allelic homologous loci, particularly between transposable elements, is a major underlying mechanism of chromosomal rearrangements [64]. The three Ascomycota subphyla display sharp differences in the abundance of transposable elements. However, as above-mentioned, the different abundance of TEs is unlikely a leading factor because the numbers of TEs are also quite different among different fungal species within a subphylum [62]. Therefore, it remains largely unclear about why the Pezizomycotina species have significantly higher rates of genome rearrangement than the other two lineages.

The second question is that how chromosomal rearrangements were fixed in populations considering its deleterious effect on sexual reproduction? Avelar et al. demonstrated that the deleterious effect in sexual reproduction by chromosomal rearrangements in fission yeast could be compensated by a strong growth advantage in asexual reproduction, the dominant form in yeasts, in certain environments [12]. Thus, the fixation of chromosomal rearrangements can be promoted in a local population [65]. Furthermore, the natural life cycle of budding yeasts with one sexual cycle only every 1000 asexual generations [66], which makes them particularly susceptible to random drift. The genomes of budding yeast have undergone repeated bottleneck due to the expansion of local populations [67]. Therefore, we speculate that the fixation of chromosomal rearrangements by random drift may serve as a mechanism to facilitate species diversification. This hypothesis can be tested by future studies using experimental evolution approaches.

## Conclusions

Based on comparative analysis of genomes of 71 species and 216 strains in Ascomycota, we found that rates of genome rearrangement are highly heterogeneous among Ascomycota lineages. The rates of genome rearrangement positively correlate with species richness in both ranks of subphylum and class. Furthermore, our data suggest that the different rates of imbalanced rearrangement, such as deletions, are a major contributor to the heterogeneous rearrangement rates. This study supports that a higher rate of genome rearrangement at the genome scale might have accelerated the speciation process and increased species richness during the evolution of Ascomycota species. Our findings provide a plausible explanation for the species richness disparity among Ascomycota lineages, which will be valuable to unravel the underlying causes for the species richness disparity in many other taxonomic groups.

## Methods

### Data source

The genomic sequences, protein sequences and genome annotation of fungal species examined were retrieved from the NCBI Reference Sequence Database (RefSeq) (Additional file 1: Table S1). Raw reads and genome assemblies for 155 *S. cerevisiae* strains were obtained from Gallone et al. [68]. Raw sequencing reads of 32 *Sch. pombe* and 29 *N. crassa* strains were downloaded from the NCBI SRA database (Additional file 7: Table S5).

### Identification of orthologous groups and phylogenetic inference of species tree

Pairwise orthologous groups between two species were identified using InParanoid 8 [39]. We identified 160 sets of 1:1 orthologous protein groups from 71 Ascomycota species and a Basidiomycota species *Ustilago maydis*, which was used as an outgroup (Additional file 2: Table S2). The 1:1 orthologous protein group here was defined as a gene family that only contains a single copy in each of the 72 species. Multiple sequence alignments were generated using MUSCLE [69]. The poorly aligned regions were further trimmed using trimAl v1.2 [70]. A maximum likelihood (ML) analysis was performed for each of the 160 orthologous groups using RAxML v8.2.10 with 100 bootstrap replicates [71] under PROTGAMMAIJTTF model as recommended by ProtTest.3.4.2 [72]. Phylogenetic reconstruction was performed with all gene sets using the coalescence method implemented in ASTRAL v5.5.6 [73]. The genetic distance between two species was calculated based on the sequence alignment concatenated from the 160 alignments using PHYLIP [74] with Jones-Taylor-Thornton (JTT) substitution model (Additional file 3: Table S3).

### Quantifying gene order divergence

To calculate the divergence of gene order, we first assign a number to each gene based on their coordination from 5’end to 3’end on each chromosome. Specifically, the genome coordination of gene *i* and *j* in the same chromosome of species A is denoted as *L*_*Ai*_ and *L*_*Aj*_, respectively. For example, the first and second gene located on chromosome 1 of species A are given genome coordination *L*_*A1*_ = 10,001 and *L*_*A2*_ = 10,002. If *L*_*Ai*_ and *L*_*Aj*_ are neighboring genes, their gene order distance *D*_*ij*_ in species A is calculated as the absolute number of genome coordination differences *D*_*Aij*_ = | *L*_*Ai*_ – *L*_*Aj*_ | = 1. Similarly, the gene order of the orthologs of gene *i* and *j* in species B (*D*_*Bij*_)is calculated as | *L*_*Bi*_ – *L*_*Bj*_ |. Therefore, if the threshold to define a conserved gene order is *D*_*ij*_ = 1, and *D*_*Bij*_ = 1, the gene order of *i* and *j* between species A and B is considered as conserved (*c*_*ij*_ = 1). If *D*_*Bij*_ > 1, their gene order is considered divergent or lost (*c*_*ij*_ = 0). As different conservation thresholds (*D*_*ij*_ = 1 ~ 5) have been examined and similar patterns were observed. Thus, we only present the results based on threshold of *D*_*ij*_ = 1. The proportion of gene order divergence (*pGOD*) between two genomes was calculated as the ratio of lost gene neighborhood among all gene neighborhoods:

1 $$ pGOD=1-\frac{\Sigma {c}_{ij}}{\left({N}_1+{N}_2-{n}_1-{n}_2\right)/2}, $$

where *N*_*1*_ and *N*_*2*_ are the numbers of genes of the two genomes examined, and *n*_*1*_ and *n*_*2*_ represent the numbers of chromosomes in the two genomes.

Although the loss of gene neighborhood occurred under a very low rate per generation, multiple breakages in the same gene neighborhood might have occurred if the divergence time between two species is sufficiently long. Moreover, the rates of gene order divergences are heterogeneous across different chromosomal regions. The probability of occurrence of a gene order divergence at a given neighborhood follows the gamma distribution. Therefore, the gamma distance of gene orders *dGOD* can be estimated by Eq. 2:

2 $$ dGOD=\alpha \left[{\left(1- pGOD\right)}^{-1/\alpha }-1\right], $$

where *α* is the shape or gamma parameter. The *α* values were estimated based on the distribution of *pGOD* values of all chromosomal regions. Specifically, we used a sliding-window analysis to obtain the *pGOD* values of all chromosomal regions between two genomes. To mitigate large variations due to small sample size, we used a window size of 50 genes and moved by every 25 genes. The *α* value was then calculated using the MASS package in R (Additional file 6: Table S4).

### Sequencing read processing, genome assembly, and estimation of genetic distances between genomes

We assessed the quality of the raw reads using FastQC v0.11.3 (https://www.bioinformatics.babraham.ac.uk/projects/fastqc/). BBtools v35.51 (http://jgi.doe.gov/data-and-tools/bbtools/) was used to filter reads with low-quality bases. Both read-ends were trimmed by 5 bp. 3′-ends were trimmed until there were at least 5 consecutive bases with quality above 20. We filtered any reads with average quality below 20, more than 3 uncalled bases, or length shorter than 50 after trimming. De novo assembly of each strain’s genome was carried out using SPAdes v3.6.2 [75]. We only used strains with sequencing coverage higher than 50X (Additional file 7: Table S5). Genetic distance (Additional file 7: Table S5) between each strain and the reference genome of respective species was estimated from genome assembly using Mash v1.1.1 [76].

### Identification and validation of structural variations based on paired-end sequencing data

Paired-end reads were aligned to the reference genomes using BWA-MEM v0.7.15 [77]. Only uniquely-mapped reads, defined here as having mapping quality above 20, were used. Initial structural variant (SV) were identified using GRIDSS v1.4.0 [78], which utilizes local-assembly, split-read, and read-depth evidence. SV calls with one or more of the following criteria were filtered: size less than 100 bp, GRIDSS quality score less than 1000, left end not assembled, right end not assembled, or within 30 kbp of a telomeric or centromeric region. Because many deletions and insertions only included transposable elements, we also filtered deletion, insertion, inversion and duplication calls that had 90% or more reciprocal overlap with a transposable element using BEDtools v2.26.0 [79] and a custom script.

To further filter false positive SV calls and delineate breakpoints, we performed local assembly for all candidate SVs, inspired by Malhotra et al. [80]. Read pairs within 1 kbp of candidate breakpoints were extracted using SAMtools v1.3.1 [81] and re-synchronized using a custom script. De novo assembly of breakpoint-spanning contigs was performed using the overlap-based (OLC) assembler Fermi-lite [82], considering the number of reads in a 2 kbp window can be relatively small. Contigs were aligned to the reference using YAHA v0.1.83 [83], which is optimized for finding spilt-alignments. Split-alignments were allowed 75% of overlap in the contig. SV validity was then inferred from the alignment results. A deletion was considered valid if the distance between split-alignments was larger in the reference than in the contig by at least 100 bp. Similarly, an insertion was considered valid if the distance between split-alignments was larger in the contig than in the reference by at least 100 bp. An inversion was considered valid if a sequence larger than 100 bp aligned to its reverse complement. A duplication was judged valid if split-alignments had a 100 bp larger overlap in the reference than their overlap in the contig. A translocation was judged valid if split-alignments came from two different chromosomes. Secondary alignments were considered when validating duplications and translocations (YAHA parameter “-FBS Y”). For deletions, insertions and tandem duplications, we required that breakpoints reported by local assembly overlap within +/− 100 bp of GRIDSS breakpoints. For translocations, we required that one breakpoint reported by local assembly overlaps with a GRIDSS breakpoint +/− 100 bp, and that the other breakpoint reported by local assembly be from the same chromosome of the other GRIDSS breakpoint.

## Additional files

Additional file 1: **Table S1.** List of species examined in this study and genome assembly version. (XLSX 16 kb)

Additional file 2: **Table S2.** List of genes in 160 orthologous groups identified in this study. (XLSX 134 kb)

Additional file 3: **Table S3.** Raw data of gene order divergence and sequence distance. (XLSX 294 kb)

Additional file 4: **Figure S1.** Examples of significant variation of *pGOD* among different chromosomal regions in *Saccharomyces cerevisiae*, *Schizosaccharomyces pombe* and *Neurospora crassa*. A sliding-windows analysis was performed to calculate the *pGOD* values among different chromosomal regions. Each window includes 50 genes and moves by every 25 genes. (PNG 240 kb)

Additional file 5: **Figure S2.** Examples of distribution of *pGOD* values in *Saccharomyces cerevisiae*, *Schizosaccharomyces pombe* and *Neurospora crassa*. The α value was calculated using the MASS package in R. (PNG 80 kb)

Additional file 6: **Table S4.** Gamma parameters estimated in all pairwise comparison between *Saccharomyces cerevisiae*, *Schizosaccharomyces pombe* and *Neurospora crassa* and other species. (XLSX 11 kb)

Additional file 7: **Table S5.** List of sequencing data information, assembly, and genetic distance for 216 strains in *Saccharomyces cerevisiae*, *Schizosaccharomyces pombe* and *Neurospora crassa*. (XLSX 25 kb)

Additional file 8: **Table S6.** List of structural variants identified from the genome sequencing data of 216 strains. (XLSX 2424 kb)

## Abbreviations

GOD: Gene order divergence
WGD: Whole genome duplication
