# Supplementary figures and images for: Heterogeneous rates of genome rearrangement contributed to the disparity of species richness in Ascomycota
Source: BMC Genomics. 2018 Apr 24;19:282. doi: 10.1186/s12864-018-4683-0 (PMC5937819; doi:10.1186/s12864-018-4683-0)

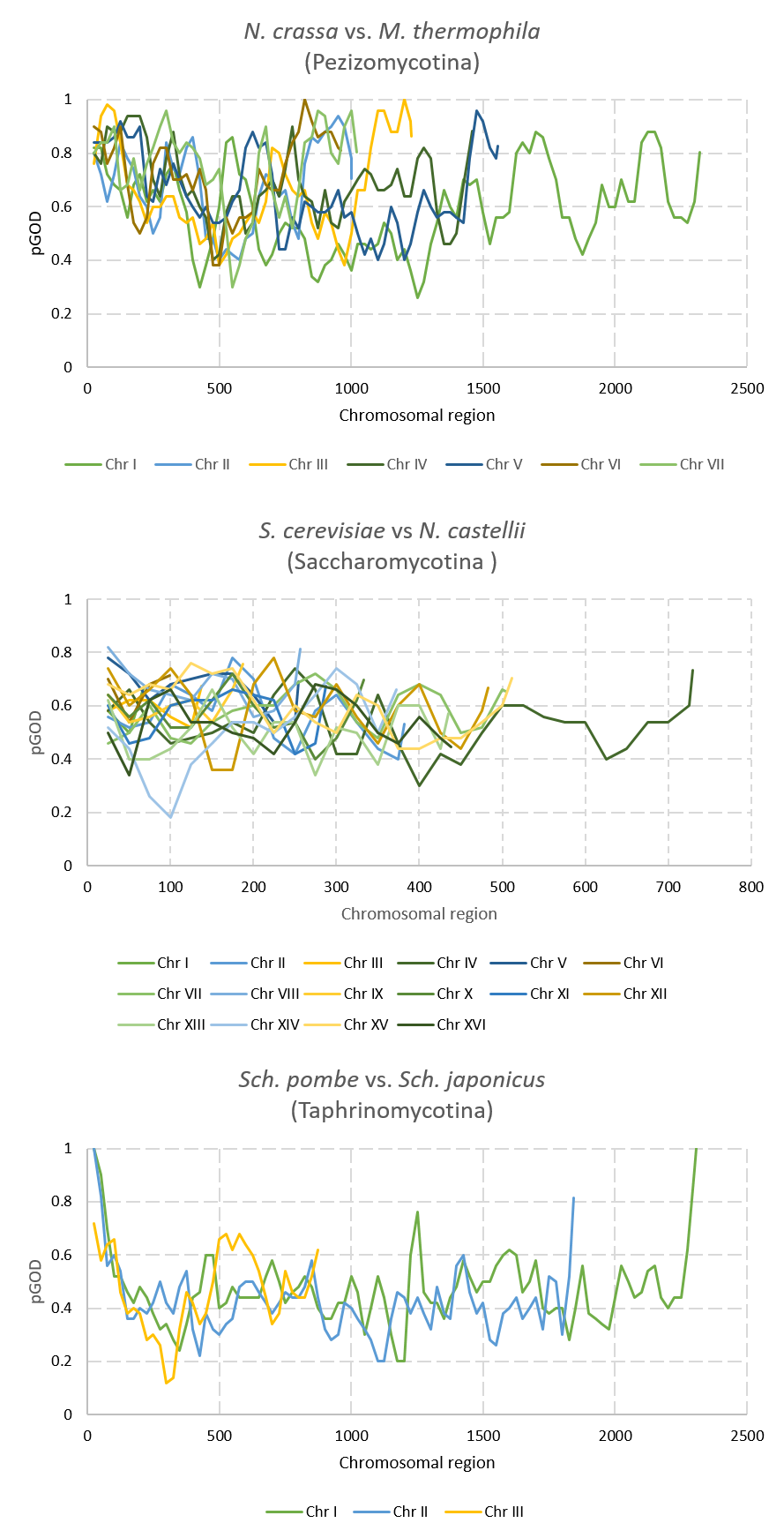

Supplement: Supplementary file 4 — Figure S1. Examples of significant variation of pGOD among different chromosomal regions in Saccharomyces cerevisiae, Schizosaccharomyces pombe and Neurospora crassa. A sliding-windows analysis was performed to calculate the pGOD values among different chromosomal regions. Each window includes 50 genes and moves by every 25 genes. (PNG 240 kb) [file 12864_2018_4683_MOESM4_ESM.png]

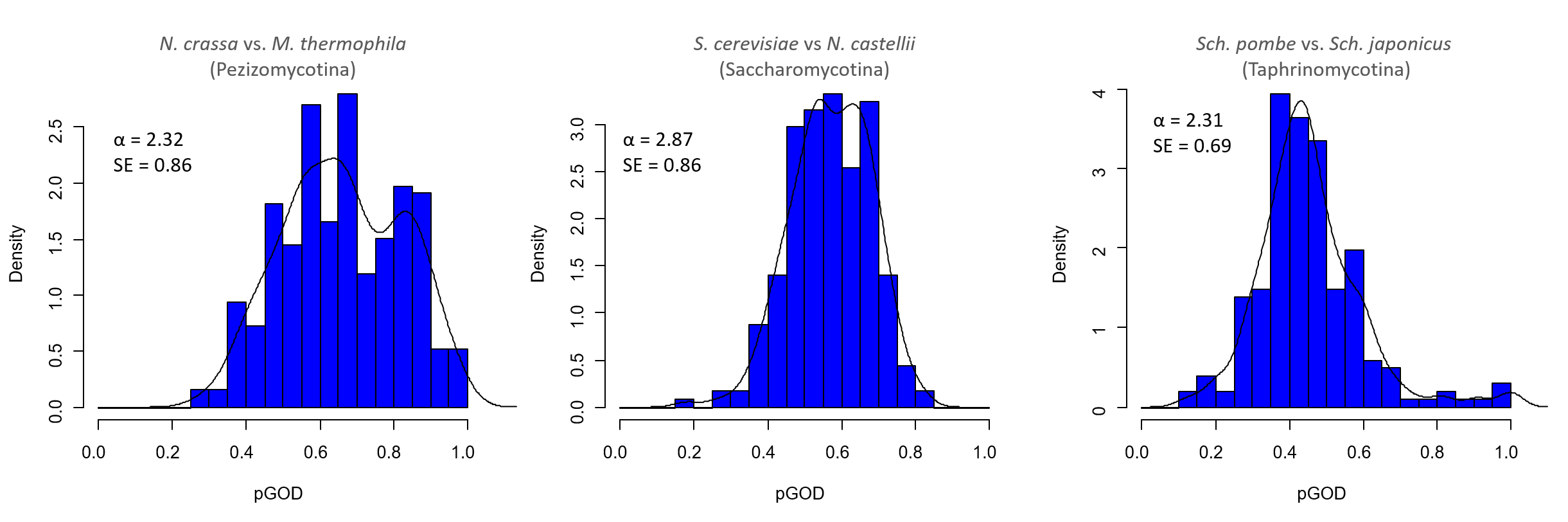

Supplement: Supplementary file 5 — Figure S2. Examples of distribution of pGOD values in Saccharomyces cerevisiae, Schizosaccharomyces pombe and Neurospora crassa. The α value was calculated using the MASS package in R. (PNG 80 kb) [file 12864_2018_4683_MOESM5_ESM.png]
